# The Evolution of Self-Fertile Hermaphroditism: The Fog Is Clearing

**DOI:** 10.1371/journal.pbio.0030030

**Published:** 2004-12-28

The nematode Caenorhabditis elegans is a little less lonely than the rest of us—it is a self-fertile hermaphrodite, which as a larva makes and stores sperm before switching to egg production for the remainder of its lifespan. (C. elegans also maintains some males at a low frequency, about 1 in 500, and the hermaphrodite's eggs can be fertilized by sperm either from males or themselves.) A sister species, C. briggsae, is also hermaphroditic, but phylogenetic evidence suggests the last common ancestor of the two species had a female/male mode of reproduction. This raises the question of how the sex determination mechanisms, which must have evolved independently, differ between the two species. In this issue, Sudhir Nayak, Johnathan Goree, and Tim Schedl show that a crucial difference lies in the activities of two genes.

In C. elegans, the early period of sperm production is controlled by multiple proteins, two of which are the focus of this study, the RNA-binding protein GLD-1 (encoded by the gene *gld-1*) and the F-box-containing protein FOG-2 (encoded by the gene *fog-2*). Together, they repress translation of a gene, *tra-2*, by binding to its messenger RNA. This allows another gene, *fem-3*, to transiently masculinize the larval germline to produce sperm.[Fig pbio-0030030-g001]

**Figure pbio-0030030-g001:**
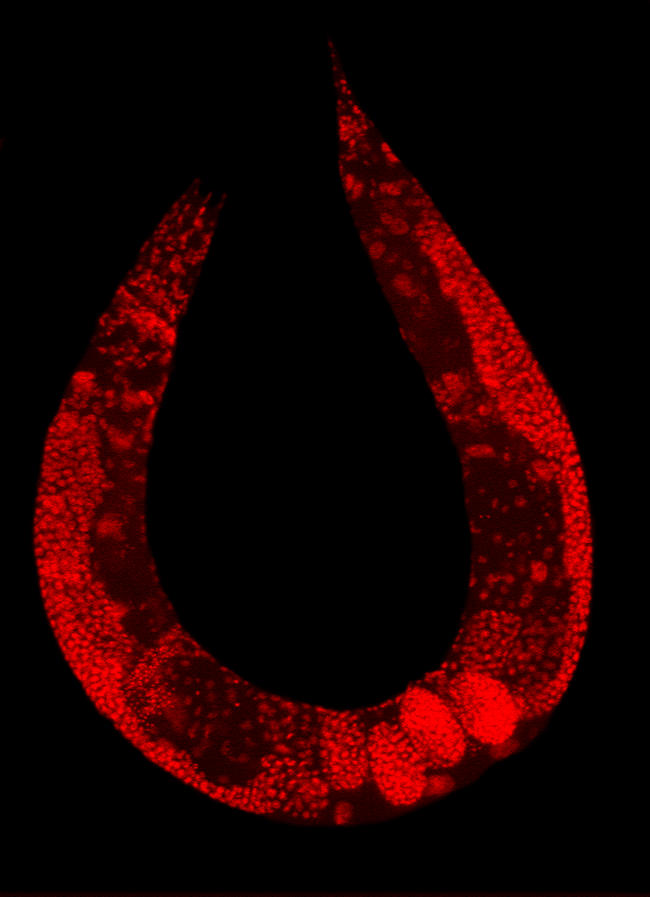
Wild-type C. elegans hermaphrodite stained to highlight the nuclei of all cells

Comparing the genomes of C. elegans and C. briggsae, Schedl and colleagues found they share 30 out of 31 sex determination genes, but not *fog-2*. More surprisingly, they found that the role of *gld-1* in sex determination is opposite in the two species. When C. elegans is deprived of *gld-1*, would-be hermaphrodites produce only oocytes. But when C. briggsae is deprived of *gld-1*, would-be hermaphrodites produce only sperm. Thus, the authors conclude, the control of hermaphrodite spermatogenesis is fundamentally different in the two species.

By further examining the C. elegans genome, the authors showed that *fog-2* arose from a gene duplication event after the C. elegans–C. briggsae split, which occurred approximately 100 million years ago. Since then, its final exon, which codes for the C-terminal end of the protein, has undergone rapid evolution. The authors also show that this is the “business end” of the protein for its interaction with GLD-1, suggesting that the divergence of C. elegans and C. briggsae sex determination pathways resulted, in part, from FOG-2's new interaction with GLD-1.

Exactly what the role of *fog-2* is in C. elegans is still unclear. The authors speculate that it may recruit additional factors onto the *gld-1/tra-2* mRNA complex, increasing efficiency of translation repression. Much remains to be discovered about C. briggsae sex determination as well. The authors suggest that additional genetic differences promoting self-fertility are likely to have accumulated since the two species diverged, which may act to strengthen the male–female germline switching signal. Investigation of this possibility may shed more light on how hermaphroditism operates in these two species, and how a developmental pathway controlling sex determination can evolve.

